# Commentary: The relationship between stress and academic burnout in college students: evidence from longitudinal data on indirect effects

**DOI:** 10.3389/fpsyg.2025.1743668

**Published:** 2026-01-13

**Authors:** Aika Suzuki

**Affiliations:** Independent Researcher, Melbourne, VIC, Australia

**Keywords:** burnout, fragile self-esteem, secure self-esteem, self-esteem, support, unconditional positive regard, wellbeing

## 1 Introduction

Zhang et al. (2025) found that higher stress increases burnout and reduces perceived social support. Their Dual Buffering Pathway Model of Academic Burnout showed that self-esteem (an internal resource) and social support (an external resource) independently mediated the relationship between stress and academic burnout and sequentially mediated this relationship.

While the study exhibited methodological rigor, the conceptualization of self-esteem and social support could be further refined. Specifically, the study treated self-esteem as a single construct rather than distinguishing secure from fragile forms, and it assessed social support without considering its quality or conditionality.

This commentary therefore advances two theoretical expansions. First, it argues that differentiating between secure and fragile self-esteem offers a more precise explanation for variability in academic burnout outcomes. Second, it proposes that identifying unconditionality in social support provides insight into a theoretical shift from its quantity to its quality in reducing burnout risks. Integrating these dimensions would enhance both the explanatory depth and the practical implications of Zhang et al. (2025)'s findings.

## 2 Reassessing self-esteem: secure vs. fragile pathways to burnout

Zhang et al. (2025) viewed self-esteem as a burnout buffer, yet their own results showed that higher self-esteem sometimes predicted greater burnout. This inconsistency invites a reconsideration of the quality of self-esteem rather than its level. Although Zhang et al. (2025) acknowledged that this unexpected result may reflect unstable self-esteem, their model did not explicitly capture this distinction. This limitation stems from the original dataset's lack of indicators of self-esteem contingency; the self-esteem scale they used (Rosenberg, 1965) measures greater or lower levels of self-esteem, not its type.

Contemporary self-esteem theory distinguishes secure self-esteem, marked by stable self-worth, from fragile self-esteem, which is contingent on achievement or approval (Crocker and Wolfe, 2001; Deci and Ryan, 1995; Kernis and Goldman, 2006; Rogers, 1951). Secure self-esteem supports resilience, whereas fragile self-esteem heightens sensitivity to failure and increases burnout risk (Freudenberger and Richelson, 1981). In academic settings, an empirical study showed that contingencies of self-worth predict academic difficulties experienced by college freshmen beyond the level of self-esteem (Crocker and Luhtanen, 2003).

Applying this distinction between secure and fragile self-esteem, the unexpected positive link between self-esteem and burnout in Zhang et al. (2025) can be reinterpreted: participants with fragile self-esteem may have scored high on the Rosenberg's self-esteem scale (1965), yet their self-worth was contingent upon academic success. This form of “fragile high self-esteem” often drives overcommitment, perfectionism, and vulnerability to burnout (Freudenberger and Richelson, 1981). Incorporating this differentiation into future models would clarify why self-esteem may, under certain conditions, act as a risk factor rather than a protective buffer.

## 3 The overlooked role of unconditional social support

The authors demonstrated that social support negatively predicts academic burnout and positively predicts self-esteem (Zhang et al., 2025). Yet, the construct of social support is treated primarily as a quantitative resource. This view neglects its qualitative dimension, particularly the distinction between conditional and non-conditional support.

Research on unconditional positive regard showed that affirming individuals' worth regardless of their achievements helps cultivate secure self-esteem (Rogers, 1951). In academic settings, an empirical study showed that both parental and teacher conditional regard—where affection depends on academic performance—contribute to school burnout in adolescents, mainly by fostering academic contingent self-esteem (Lavrijsen et al., 2023). This suggests that certain forms of support play a crucial role in predicting burnout risk. However, Zhang et al. (2025) used the Social Support Scale (Xiao, 1994), which does not assess unconditional support. A higher score simply reflects more support (Xiao, 1994), not whether that support was unconditional. Because its three dimensions (objective support, subjective support, and support utilization) can be offered either conditionally or unconditionally, the measure cannot distinguish between these forms. Therefore, Zhang et al. (2025)'s findings could not address the role of support conditionality in the stress–burnout process.

Integrating the dimension of unconditional positive regard could significantly strengthen its explanatory and practical power of Zhang et al.'s (2025) findings. Theoretically, it clarifies which types of support enhance secure self-esteem and thereby mitigate academic burnout risk. Practically, it helps identify which forms of support are most effective for reducing students' vulnerability to academic burnout.

## 4 Toward a refined dual buffering path model of academic burnout

A refined interpretation of the “Dual Buffering Pathway Model of Academic Burnout” can thus be proposed:

- Internal buffering (Self-Esteem) functions through secure rather than fragile self-esteem, which enables students to tolerate stress without overidentifying with performance outcomes.
- External buffering (Social Support) operates not merely through the presence of social support, but through its non-conditionality—a factor that fosters internal stability.

In a refined buffering model, non-conditional social support would enhance secure self-esteem, which then mediates the effect of stress on burnout.

Operationalizing these constructs requires more targeted measurement. Future studies could employ multidimensional self-esteem measures, such as the Contingent Self-Esteem Scale (Kernis and Goldman, 2006), to capture secure vs. fragile self-esteem. Additionally, developing a non-contingent support measure or incorporating qualitative assessments would clarify distinctions between contingent and non-contingent support. Qualitative assessments can be done through in-depth interviews to understand these dynamics in a real-life setting. Those scales and in-depth interviews would enable a nuanced analysis of how fragile vs. secure self-worth interacts with perceived support to influence burnout risks.

## 5 Discussion

Zhang et al.'s (2025) study can be strengthened by integrating different self-esteem and social support theories. Future research should distinguish secure from fragile self-esteem and assess support conditionality to clarify their buffering effects.

Zhang et al. (2025) argued that reducing academic burnout requires shifting from an exclusive focus on achievement to supporting students' holistic development. Such unconditional support may foster secure self-esteem, thereby reducing burnout risks, as seen in the refined buffering model. However, without further investigation, specific initiatives should not be implemented because no form of self-esteem is inherently superior, and individuals can thrive with either type. While secure self-esteem protects against burnout, it is also true that those with fragile self-esteem are often high achievers due to their relentless pursuit of excellence (Freudenberger and Richelson, 1981).
